# Supplementary material for: Causal Relationship between Aging and Anorexia Nervosa: A White-Matter-Microstructure-Mediated Mendelian Randomization Analysis
Source: Biomedicines. 2024 Aug 16;12(8):1874. doi: 10.3390/biomedicines12081874 (PMC11351342; doi:10.3390/biomedicines12081874)
Supplement: Supplementary file 1 [file biomedicines-12-01874-s001.zip › Supplementary Figure/Figure S4/Singe SNP plot of MR analysis for TL to ubm-b-1839.pdf]

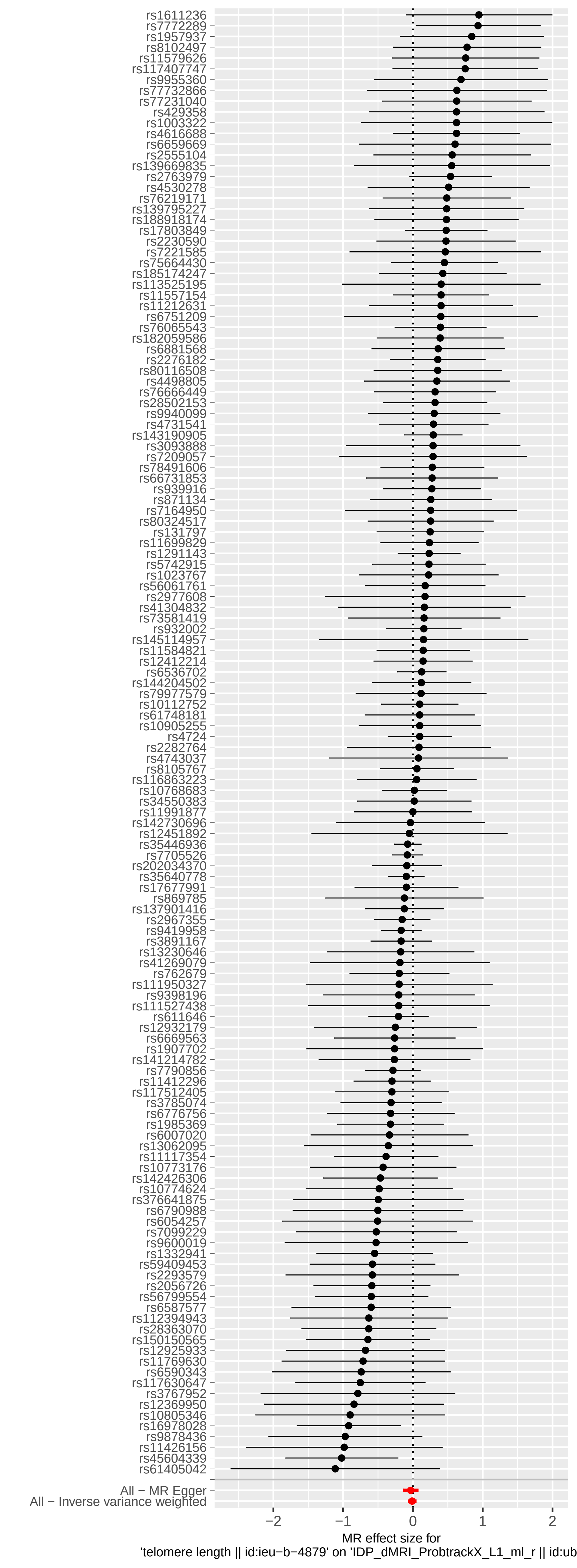
rs1611236
rs7772289
rs1957937
rs8102497
rs11579626
rs117407747
rs9955360
rs77732866
rs77231040
rs429358
rs1003322
rs4616688
rs6659669
rs2555104
rs139669835
rs2763979
rs4530278
rs76219171
rs139795227
rs188918174
rs17803849
rs2230590
rs7221585
rs75664430
rs185174247
rs113525195
rs11557154
rs11212631
rs6751209
rs76065543
rs182059586
rs6881568
rs2276182
rs80116508
rs4498805
rs76666449
rs28502153
rs9940099
rs4731541
rs143190905
rs3093888
rs7209057
rs78491606
rs66731853
rs939916
rs871134
rs7164950
rs80324517
rs131797
rs11699829
rs1291143
rs5742915
rs1023767
rs56061761
rs2977608
rs41304832
rs73581419
rs932002
rs145114957
rs11584821
rs12412214
rs6536702
rs144204502
rs79977579
rs10112752
rs61748181
rs10905255
rs4724
rs2282764
rs4743037
rs8105767
rs116863223
rs10768683
rs34550383
rs11991877
rs142730696
rs12451892
rs35446936
rs7705526
rs202034370
rs35640778
rs17677991
rs869785
rs137901416
rs2967355
rs9419958
rs3891167
rs13230646
rs41269079
rs762679
rs111950327
rs9398196
rs111527438
rs611646
rs12932179
rs6669563
rs1907702
rs141214782
rs7790856
rs11412296
rs117512405
rs3785074
rs6776756
rs1985369
rs6007020
rs13062095
rs11117354
rs10773176
rs142426306
rs10774624
rs376641875
rs6790988
rs6054257
rs7099229
rs9600019
rs1332941
rs59409453
rs2293579
rs2056726
rs56799554
rs6587577
rs112394943
rs28363070
rs150150565
rs12925933
rs11769630
rs6590343
rs117630647
rs3767952
rs12369950
rs10805346
rs16978028
rs9878436
rs11426156
rs45604339
rs61405042
All − MR Egger
All − Inverse variance weighted
−2
−1
0
1
2
MR effect size for
'telomere length || id:ieu−b−4879' on 'IDP_dMRI_ProbtrackX_L1_ml_r || id:ub
